# A junctionless SONOS nonvolatile memory device constructed with *in situ*-doped polycrystalline silicon nanowires

**DOI:** 10.1186/1556-276X-7-162

**Published:** 2012-02-29

**Authors:** Chun-Jung Su, Tuan-Kai Su, Tzu-I Tsai, Horng-Chih Lin, Tiao-Yuan Huang

**Affiliations:** 1 Nano Facility Center, National Chiao Tung University, Hsinchu, 300, Taiwan; 2 Department of Electronics Engineering and Institute of Electronics, National Chiao Tung University, Hsinchu, 300, Taiwan; 3 Department of Electrophysics, National Chiao Tung University, Hsinchu, 300, Taiwan; 4 National Nano Device Laboratories, 26 Prosperity Road I, Hsinchu, 300, Taiwan

**Keywords:** JL; NW, poly-Si, SONOS, TFT

## Abstract

In this paper, a silicon-oxide-nitride-silicon nonvolatile memory constructed on an n^+^-poly-Si nanowire [NW] structure featuring a junctionless [JL] configuration is presented. The JL structure is fulfilled by employing only one *in situ *heavily phosphorous-doped poly-Si layer to simultaneously serve as source/drain regions and NW channels, thus greatly simplifying the manufacturing process and alleviating the requirement of precise control of the doping profile. Owing to the higher carrier concentration in the channel, the developed JL NW device exhibits significantly enhanced programming speed and larger memory window than its counterpart with conventional undoped-NW-channel. Moreover, it also displays acceptable erase and data retention properties. Hence, the desirable memory characteristics along with the much simplified fabrication process make the JL NW memory structure a promising candidate for future system-on-panel and three-dimensional ultrahigh density memory applications.

## Introduction

With the proliferation of portable electronic products, the demand of high density nonvolatile memories [NVMs] has boosted tremendously. Among various nonvolatile memory [NVM] architectures, the flash memory, based primarily on floating-gate [FG] devices, has dominated the mainstream NVM market for decades. FG devices, however, are inherently vulnerable to fatal data loss through a single defect in the tunnel oxide [1], and face stringent challenges in the course of device downscaling owing to the gate-coupling concern [2]. In light of this, flash memory based on charge trapping [CT] devices, such as silicon-oxide-nitride-oxide-silicon [SONOS] multilayer structure [3] and its various derivatives [4,5], has received renewed interest, and is extensively investigated recently. Being inherently immune to gate-coupling issue and more tolerant to the defects in the thin tunnel oxide, a SONOS memory device enables thinner gate stack height for stronger electrostatic control, and thus is more scalable. Concurrently, flash memory constructed on polycrystalline silicon thin-film transistors (poly-Si [TFTs]) has attracted enormous attention owing to the low-cost and low-temperature fabrication processes and its compatibility with system-on-panel [SOP] or system-on-chip integration [6,7]. In addition, a thin-film transistor [TFT]-SONOS array is also attractive for three-dimensional [3-D] multilayer stack structure for the purpose of ultrahigh memory cells density without aggressive scaling of device dimensions [8]. However, due to the grainy structure and defects associated with grain boundaries in the films, typical poly-Si TFT-based memory devices face some challenging issues, such as poor subthreshold swing [SS] and slow memory operation speed. Nevertheless, by employing nanowire [NW] channels with multiple-gated configuration in TFTs, the memory speed and subthreshold swing have been demonstrated to be significantly improved, thanks to better gate controllability and reduced defects in the small volume of NWs [9]. Recently, we have developed a junctionless [JL] poly-Si NW transistor with enhanced drive current and reduced source/drain [S/D] series resistance using *in situ *heavily doped poly-Si [10]. Such material features uniform doping concentration and is commonly used for gate electrode in the fabrication of field-effect transistors [FETs]. A JL transistor features the same doping polarity and concentration throughout the entire device, and thus alleviates the requirement of precise control of dopant distribution in the S/D regions [11]. In this work, we further apply and investigate such scheme to SONOS flash memory device for the purpose of reducing the fabrication complexity and enhancing the programming efficiency by taking advantage of the higher carrier concentration in the JL NW channels.

### Device fabrication and experiment

**Figure 1:**
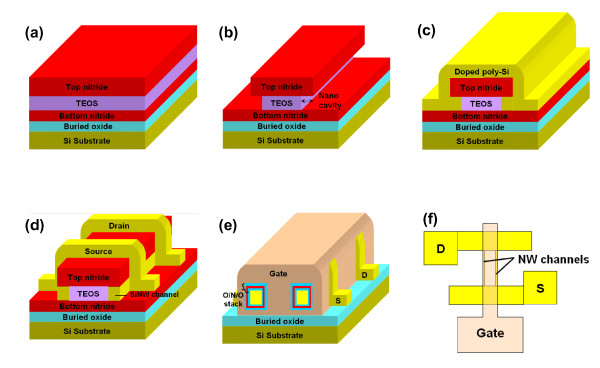
**Illustration of the key process steps for fabricating the JL NW SONOS device**. (**a**) A dielectric stack consisting of top nitride/TEOS/bottom nitride before patterning. (**b**) Formation of the nano-cavities at the two sides of the stack. (**c**) Deposition of an n^+^-doped poly-Si film. (**d**) Formation of the S/D regions and NW channels by anisotropic dry etching. (**e**) Final device structure featuring the gate-all-around configuration with an O/N/O gate dielectric stack. (**f**) Schematic top-view layout of the device.

The process flow of the proposed poly-Si NW SONOS memory device is similar to that of the previously reported NW FETs with regard to the NWs formation [10]. First, a dielectric stack consisting of top nitride/tetraethyl orthosilicate [TEOS] oxide/bottom nitride was sequentially deposited by low-pressure chemical vapor deposition [LPCVD] on a thermally oxidized Si wafer (Figure 1a). After patterning the stack by an anisotropic plasma etching, highly selective lateral etching of the TEOS oxide with diluted hydroflouric acid [HF] solution was executed to form the nanocavities at the two sides of the stack, as shown in Figure 1b. Then a 100 nm-thick *in situ *n^+^-doped poly-Si layer was deposited using SiH_4 _of 0.49 slm and PH_3 _of 15 sccm by LPCVD at 600 mtorr and 550°C (Figure 1c). The n^+^-poly-Si layer was subsequently patterned and anisotropically etched using Cl_2_/HBr gasses to define the S/D regions and NW channels to form the JL structure (n^+^-n^+^-n^+^), as illustrated in Figure 1d. It should be noted that the poly-Si embedded in the nanocavities would remain after the anisotropic etching and served as the NW channels. Before gate stack deposition, the top-nitride/bottom-nitride and TEOS oxide layers were removed by hot H_3_PO_4 _and HF solution, respectively, to expose the NW channels. Then, a gate dielectric stack of block-oxide/nitride/tunnel-oxide [ONO] with thicknesses of 12/7/3 nm was deposited by LPCVD. Next, another *in situ *phosphorous-doped n^+^-poly-Si was deposited using SiH_4 _of 0.49 slm and PH_3 _of 100 sccm by LPCVD and then patterned to serve as the gate electrode (Figure 1e). After depositing a 500 nm passivation oxide layer, standard metallization was then performed to complete the device fabrication. Figure 1f shows the schematic top-view layout of the device. It should be noted that the nominal doping concentrations are 6 × 10^20 ^cm^-3 ^and 1 × 10^20 ^cm^-3 ^for the n^+^-poly-Si gate and the NW channels, respectively. However, it has been reported that the electrical resistivity of a thin film will increase as the dimensions of the film become sufficiently small because the mean free path of conduction carriers in it is reduced [12]. Moreover, owing to the effects of donor deactivation and phosphorous segregation occurring in the Si NW structure [13,14], the effective carrier concentration in the NW channels practically would be lower than expected. In fact, we have also experimentally demonstrated that the resistivity of phosphorous-doped poly-Si NWs increases as NW's cross-sectional dimensions decrease [15].

**Figure 2:**
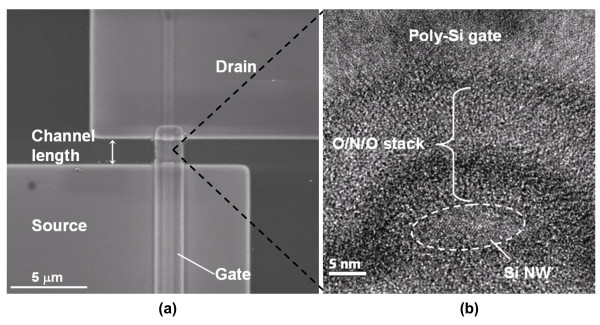
**SEM and TEM characterization of a fabricated JL poly-Si NW device**. (**a**) Top-view SEM image. (**b**) Cross-sectional TEM image of the NW channel in (a).

Figure 2a shows the top-view scanning electron microscopic [SEM] image of a fabricated JL NW device, in which the channel length is defined as the spacing between the S/D regions. The cross-sectional transmission electron microscopic [TEM] image of the NW channel is shown in Figure 2b, indicating that the cross section of the NW is about 11 × 6 nm, which is enclosed by the ONO gate stack and poly-Si gate. Ideally, the cross section of the poly-Si NWs is supposed to be rectangular in shape according to the formation scheme of NWs in this work. However, since the NWs experienced a series of wafer cleaning and etching steps conducted in chemical solutions during processing, the NW's corners were rounded, thus leading to the nearly elliptic profile as observed. In comparison, conventional inversion-mode [IM] memory cells (i.e., n^+^-i-n^+^) with undoped poly-Si NW channels having the same ONO stack were also fabricated. In this study, NW memory devices with a channel length of 0.4 μm were used for the analyses of electrical and memory characteristics.

## Results and discussion

**Figure 3:**
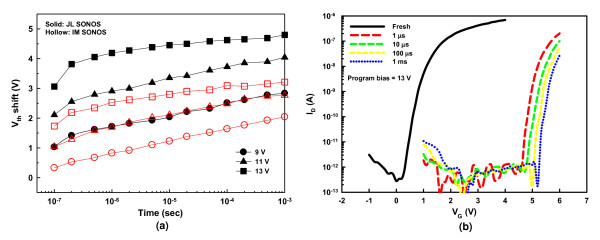
**Programming properties of the JL and IM NW SONOS devices**. (**a**) Programming characteristics at gate biases of 9, 11, and 13 V. (**b**) Evolution of the *I*_D_-*V*_G _curves for the JL device during programming at 13 V from 1 μs to 1 ms.

**Figure 4:**
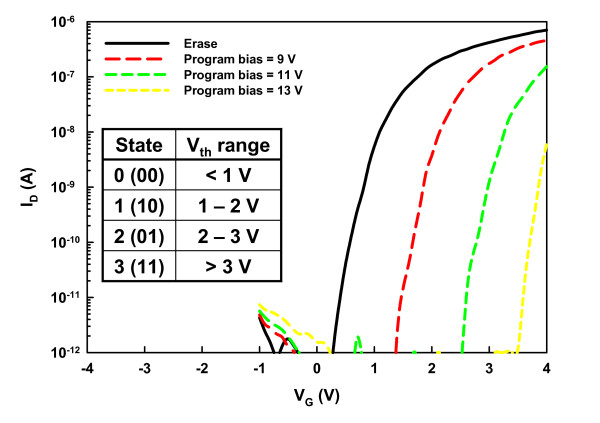
***I*_D_-*V*_G _behaviors of the JL NW device**. *I*_D_-*V*_G _behaviors of the JL NW device for multilevel programming operation with gate biases of 9, 11, and 13 V for 100 ns. Inset shows the definition of *V*_th _range for each state.

In this work, the Fowler-Nordheim [FN] tunneling mechanism was used for the program/erase [P/E] operations of the SONOS memory devices. In the programming process, large positive biases ranging from 9 to 13 V were applied to the gate while keeping the S/D grounded. Figure 3a presents the programming properties of the JL and IM SONOS memory devices. The JL memory cell apparently exhibits faster programming speed, which could be ascribed to the larger conduction-band carrier concentration in the heavily doped NW channel, thus enhancing the tunneling probability of carrier injection into the nitride trapping layer. With a larger number of electrons tunneling through the tunnel oxide and getting trapped in the nitride layer, a wider window of *V*_th _shift is resulted. Also note that a program window up to 4.8 V can be obtained without noticeable degradation of the SS, as revealed in Figure 3b, in which the *I*_D_*-V*_G _curves in the programmed states were stressed at 13 V for duration times of 1, 10, 100 μs, and 1 ms, respectively. In contrast, the maximal program window of the IM memory cell is about 3.2 V with gate bias of 13 V for 1 ms. In addition, the capability of the large program window of the JL device ensures low voltage operation and multilevel programming with a distinct programmed *V*_th _value. Figure 4 shows multilevel programming of the JL device for four states with *V*_th _difference of 1 V between each state. The device was stressed at 9, 11, and 13 V for 100 ns to the programmed states of 1, 2, and 3, respectively. This result indicates that the JL cell could be placed in one of four discrete states, as described in the inset, to achieve 2 bits/cell storage even with a short duration of 100 ns. This confirms that the abundance of carriers in the JL device lends itself nicely to promoting the programming properties in terms of lower operation voltage, higher speed and larger program window.

**Figure 5:**
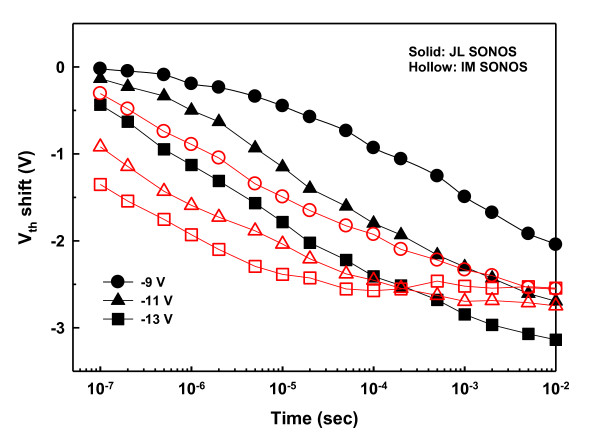
**Erase properties of the JL and IM NW SONOS cells**. Before erasing, the cells were programmed to *V*_th _shift of +3 V and +2.5 V for the JL and IM structures, respectively.

In order to study the erase behavior, the cells were firstly programmed to a memory window (Δ*V*_th_) of +3 and +2.5 V for the JL and IM devices before erasing, respectively. Negative biases were then applied to the gate with the S/D remained grounded. Figure 5 depicts the erase characteristics of the JL and IM NW SONOS cells. For SONOS CT memory devices, the erase current is composed of electron detrapping and hole injection currents, depending on the band alignment conditions. The slower erasing speed found in the JL device could be attributed to the relatively reduced hole concentration in it. However, it is of interest to note that the disparity in erasing speed between the two devices is not as remarkable as that in the programming speed. We speculate that this is due to two factors. For one, the magnitude of FN tunneling current is basically a function of the carrier effective mass and barrier height at SiO_2_/Si interface [16]. The holes have larger effective mass and higher potential barrier than the electrons used in the programming process. Hence, the reduced tunneling of holes leads to a less efficient erasing process. Besides, phosphorus impurities tend to segregate or be trapped at the surface of Si NW structures, resulting in lower effective density of the carriers at the SiO_2_/Si NW interface than that in the Si NW bulk [14]. Accordingly, a certain number of holes practically could be induced at the surface of the n^+^-poly-Si NW channels under erasing operation with large negative gate biases. This, together with the decreased tunneling of holes, may contribute to the smaller disparity in the erasing efficiency between the JL and IM memory cells. Moreover, the erasing speed of the JL device is apparently enhanced by increasing the magnitude of the gate bias. Specifically, the JL memory cell displays a comparable Δ*V*_th _of 2.5 V to that of the IM device in 0.2 ms at -13 V stress. Besides, it also shows that the erase time to achieve the memory window of 3 V is around 5 ms at -13 V, which is very desirable as compared with the recently reported data of poly-Si NW based SONOS memory cells [9,17,18]. On the other hand, a saturation behavior occurs at *t *= 10^-4 ^sec in the IM SONOS device, and beyond this, the *V*_th _values are found to slightly go up with a gate bias of -13 V, which may be attributed to the electron injection from the n^+^-poly-Si gate into the nitride traps at a high electric field. This problem could be relieved by using gate materials with higher work function such as p^+^-poly-Si, TiN, or TaN relative to the poly-Si channel [19]. Such gate materials are also conducive to obtaining a suitable *V*_th _value for heavily doped n^+^-channel devices by depletion of carriers owing to the work function difference between the channel and gate material [20].

**Figure 6:**
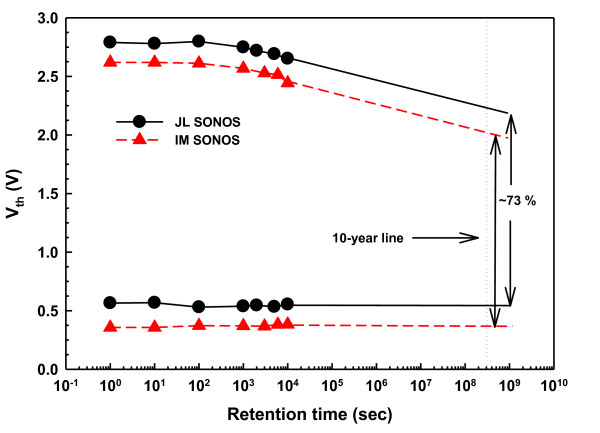
**Retention behaviors for the JL and IM NW SONOS devices at room temperature**.

Figure 6 shows the data retention characteristics of the JL and IM NW memory cells at room temperature with programming operation at 11 V for 50 μs and erasing operation at -10 V for 1 ms. Both devices reveal a charge loss of only 5% at the tested duration time of 10^4 ^s, and the extrapolated retention behavior at the end of 10 years that shows 73% of the original P/E window is maintained. Consequently, the almost identical behavior of the charge loss in both devices suggests that the carrier concentration in the channel does not exert significant difference on the data retention characteristics.

## Conclusion

In summary, we have successfully demonstrated the feasibility of JL NW SONOS memory device by employing only one *in situ*-doped n^+^-poly-Si layer. In addition to the much simplified fabrication process, the fabricated device displays enhanced programming properties and desirable data retention behavior. While no improvement in the erasing efficiency is observed, the JL device still exhibits comparable erase window to the IM counterpart. Consequently, the proposed JL NW structure with complementary metal-oxide semiconductor compatible process appears to be very promising for low-cost and ultrahigh-density NVMs for future 3-D electronics and SOP applications.

## Competing interests

The authors declare that they have no competing interests.

## Authors' contributions

CJ designed the study, carried out the SEM and TEM characterization, performed the electrical analysis and drafted the manuscript. TK and TI fabricated the samples and carried out the electrical characterization. HC and TY participated in the design and coordination of the study. All authors read and approved the final manuscript.
